# Perfecting the Craft: Composite Restoration Elevated With the Stamp Technique

**DOI:** 10.7759/cureus.69139

**Published:** 2024-09-10

**Authors:** Lalit P Pawar, Aditya Patel, Manoj Chandak, Saee Wazurkar, Mrinal Nadgouda

**Affiliations:** 1 Department of Conservative Dentistry and Endodontics, Sharad Pawar Dental College and Hospital, Datta Meghe Institute of Higher Education and Research, Wardha, IND

**Keywords:** composite, occlusal anatomy, resin composite, restoration, stamp technique

## Abstract

The "stamp technique" for posterior composite restoration placements is a relatively simple method for duplicating occlusal anatomy with near perfection. Due to the sensitivity of proprioceptors in the stomatognathic system to pressure, even slight occlusal disparities resulting from direct restorations can cause discomfort for patients. This discomfort often leads patients to adjust to a new habitual occlusal position, potentially leading to significant long-term craniomandibular issues. It was initially designed to restore Class I cavities and eroded teeth, where the marginal ridge of the tooth remains undamaged. The method is suitable for teeth with preoperative intact anatomy unaffected by carious lesions. The stamp technique aims to deliver a precise and natural-looking restoration with accurate functional occlusion. This case study demonstrates the application of the stamp technique for a straightforward Class I composite restoration, with the goal of quickly replicating the occlusal anatomy by creating a model of the original, unprepared tooth structure within minutes.

## Introduction

In the second decade of the 21st century, dentistry has undergone significant advancements, characterized by a paradigm shift towards biomimetic dentistry, a field focused on emulating natural biological processes. This evolution has led to notable improvements in both the aesthetics and functionality of dental treatments, as traditional methods are not only refined but also augmented by innovative techniques.

A key aspect of this transformation is the move away from the historical concept of "extension for prevention," which is gradually being supplanted by the principles of minimally invasive dentistry. This contemporary approach prioritizes the preservation of the tooth structure, a significant departure from earlier practices. Despite the widespread adoption of composite restorations, achieving successful aesthetic outcomes with direct composite restorations remains a challenging endeavor. These procedures are inherently technique-sensitive, requiring a high level of skill and experience to precisely replicate the natural form and occlusion of the tooth. Moreover, the time investment for finishing and polishing a composite restoration is substantially greater, often taking twice as long as an amalgam restoration [1].

Manually making an optimal direct composite restoration demands a high level of experience. The "stamp technique" stands out as a method that seamlessly combines aesthetics and function in composite restorations by accurately replicating the tooth's original anatomy based on its unprepared structure [2,3]. Dr. Waseem Riaz, a practitioner based in London, has suggested a method known as the "stamp technique" for achieving accurate occlusal topography in direct composite resin restorations. This technique has also been noted for its effectiveness in vertical bite reconstruction of dentitions that have been worn down [3].

This method involves creating an occlusal stamp that replicates the natural tooth occlusal structure before any cavity preparation. Subsequently, this stamp is constrained onto the composite restoration's final layer before curing, resulting in a faithful reproduction of the original anatomy. The technique is applicable in cases in which caries are detected during the examination of teeth exhibiting marginal ridges, which are intact, and occlusal anatomy, which is ideal. In posterior teeth, except for caries extending beyond the dentino-enamel junction, occlusal morphology is intact among the primary carious lesions. Consequently, various studies describe a restoration approach utilizing an occlusal stamp to mirror the tooth's original morphology based on its existing clinical condition, thus reducing the time essential for excess removal and restoration finishing. The stamp technique is most suitable for cases involving shallow to moderate occlusal caries where the integrity of the occlusal surface is largely intact. This ensures that the stamp can replicate the natural morphology accurately, leading to a more efficient and aesthetic restoration process. On the other hand, in situations where there is significant tooth structure loss, such as in extensive MOD (mesial-occlusal-distal) cavities or when the occlusal surface is heavily decayed and its anatomy is partially or completely missing, the stamp technique may not be appropriate. In these cases, the absence of a defined occlusal anatomy makes it difficult to create a reliable stamp, which can compromise the accuracy and quality of the restoration; for such cases, alternative restorative techniques that allow for the reconstruction of the occlusal anatomy, such as freehand composite layering or the use of preformed matrices, may be more suitable [1].

## Case presentation

**Figure 1:**
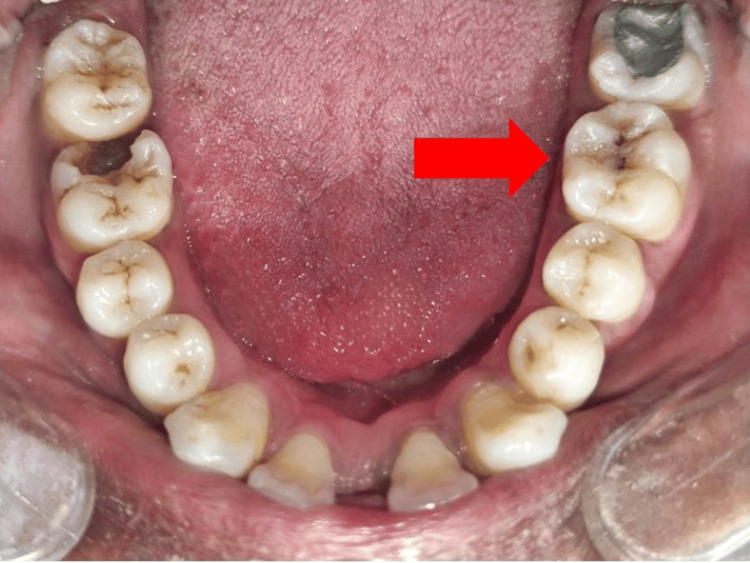
Preoperative photograph of the mandibular arch showing a decayed tooth 36

A 27-year-old male patient reported to the Department of Conservative Dentistry and Endodontics and complained of decayed teeth on his lower left back tooth region of the jaw. The patient's past medical and dental histories were unremarkable. Intraoral examination revealed pit and fissure caries on the lower left first molar, but there were no symptoms of night pain, pus discharge, or swelling. Pulpal sensitivity tests, including a thermal test, were conducted. For this test, a 0.06 #15 gutta-percha (Dentsply Sirona, Charlotte, North Carolina) was heated to 65.5°C and briefly applied to the middle third of the mesiobuccal cusp. The lower left first molar exhibited lingering pain after the stimulus was removed. To assess the extent of tooth caries, an intraoral periapical radiograph (radiovisiography) was used, showing that the marginal ridge was unaffected. The patient's diagnosis was found to be reversible pulpitis. As the marginal ridges were intact, after examination, it was decided to treat the affected tooth 36 using composite restorative material with a stamp method to restore the decayed area (Figure 1).

Treatment procedure

**Figure 2:**
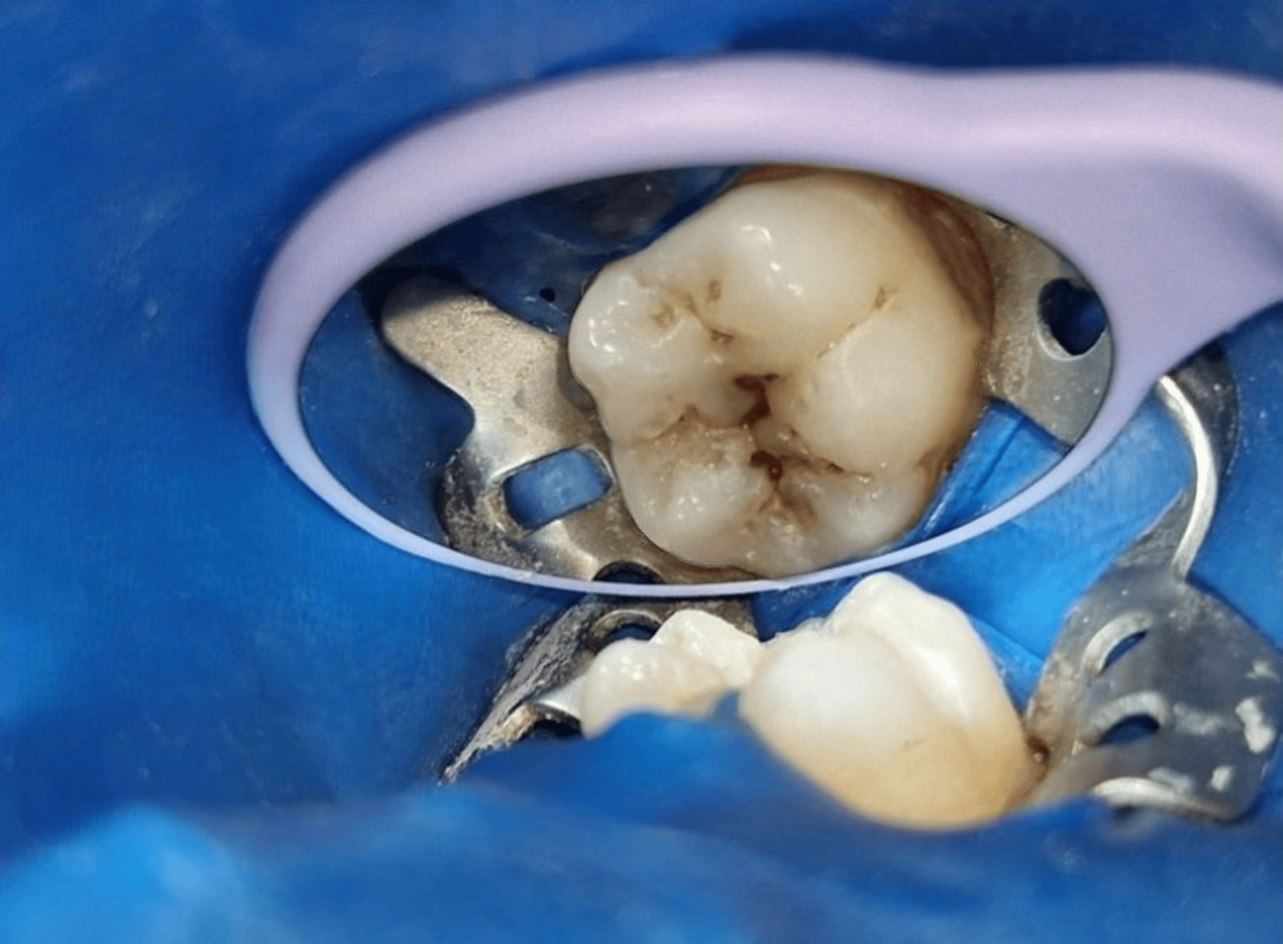
Preoperative photograph of the tooth with caries

A latex rubber dam (GDC Dental Dam Kit, Nagar, India) was used to isolate the tooth before the cavity preparation (Figure 2) [2].

**Figure 3:**
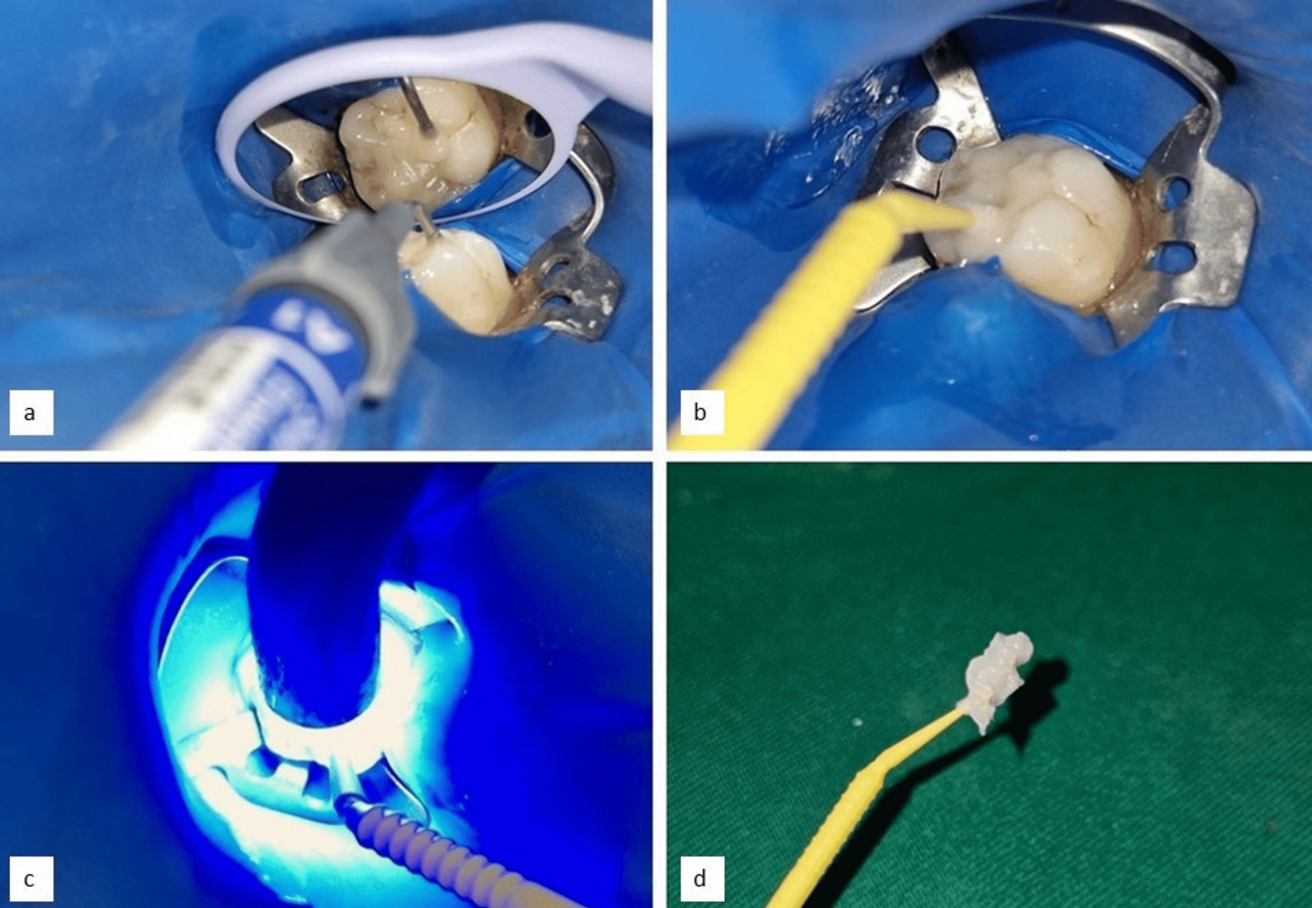
(a) Placement of the flowable composite material; (b) placement of a microbrush over the flowable composite material; (c) polymerization of the composite material with a microbrush through light cure; (d) occlusal stamp

The tooth was coated with a separating medium, specifically petroleum jelly, to facilitate the removal of the impression material later. A flowable composite (Ivoclar Tetric N-Flow, Amherst, New York) was then applied evenly to the occlusal surface of the tooth. A microbrush was gently pressed onto the flowable composite to capture the fine details of the occlusal anatomy. The composite was subsequently cured with visible light for 20 seconds, effectively creating an accurate impression of the tooth's occlusal surface. This process is illustrated in Figures 3a-d.

**Figure 4:**
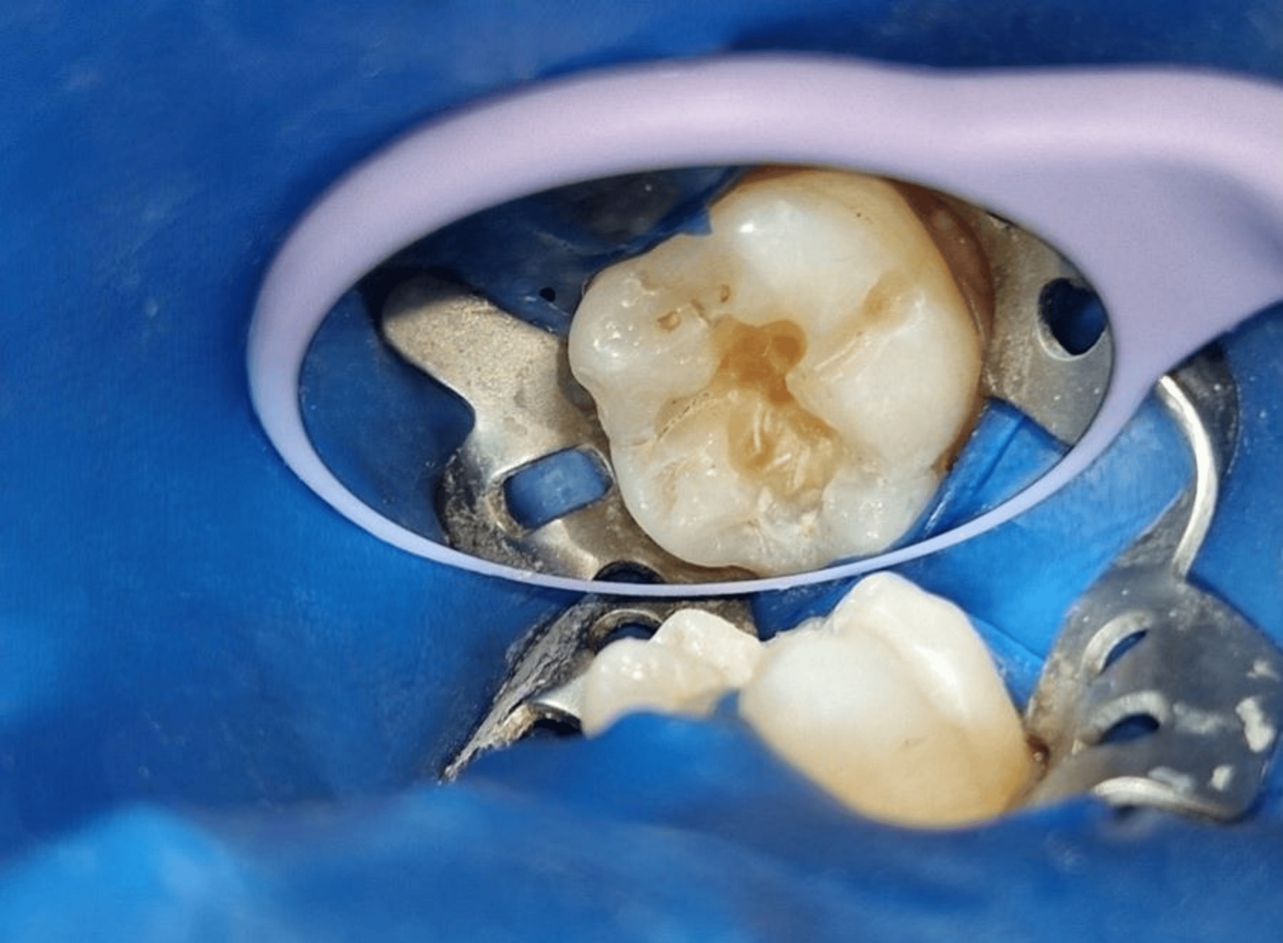
Caries excavation

A prophylactic paste was used to remove the separating media from the tooth. The caries were excavated, which led to the preparation of the Class I cavity (Figure 4).

**Figure 5:**
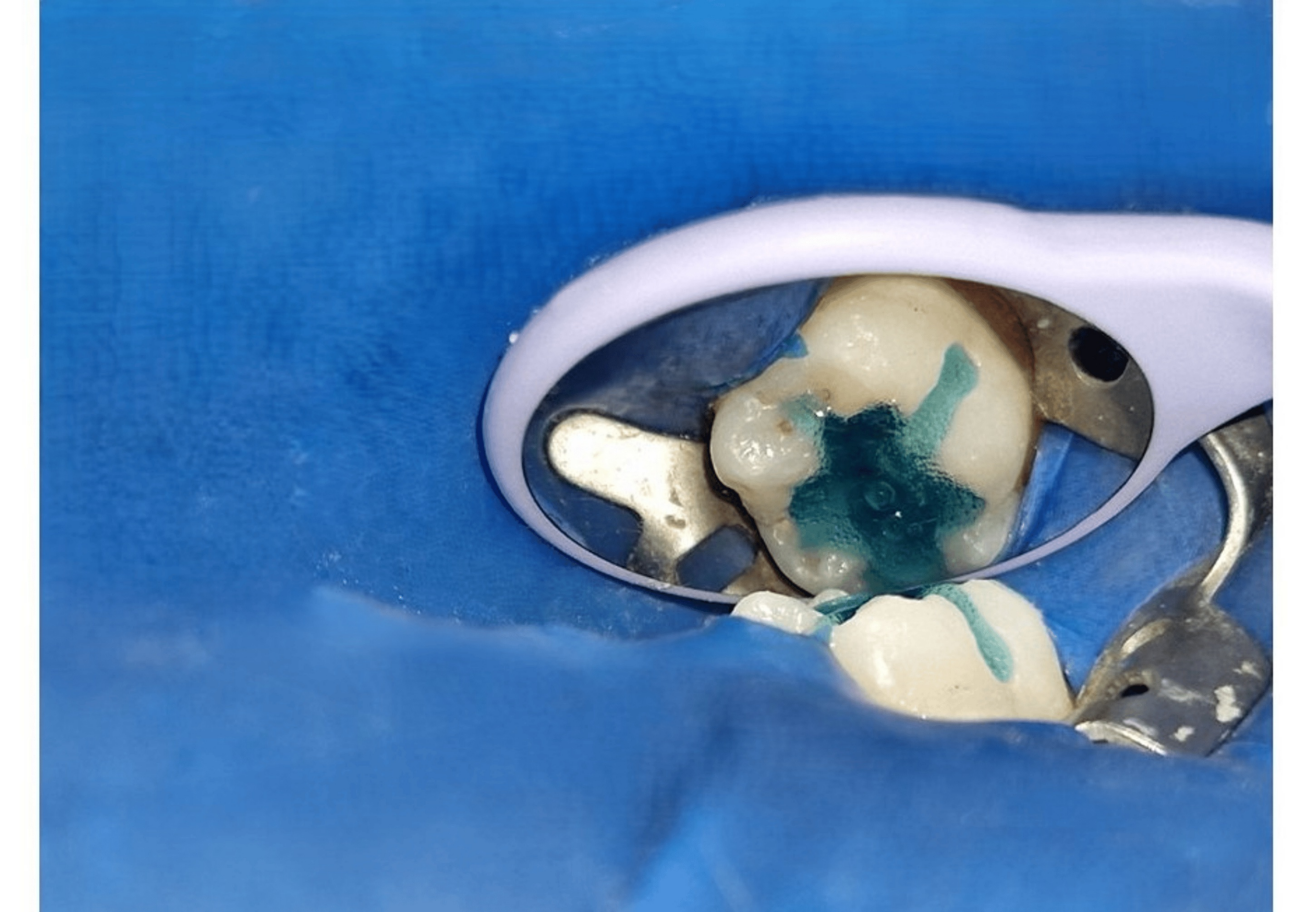
Etching using 37% phosphoric acid

The exposed dentin was treated with a 37% orthophosphoric acid etchant (Prime Dental, Thane, India) on the tooth surface for 15 seconds, then rinsed and air-dried (Figure 5).

**Figure 6:**
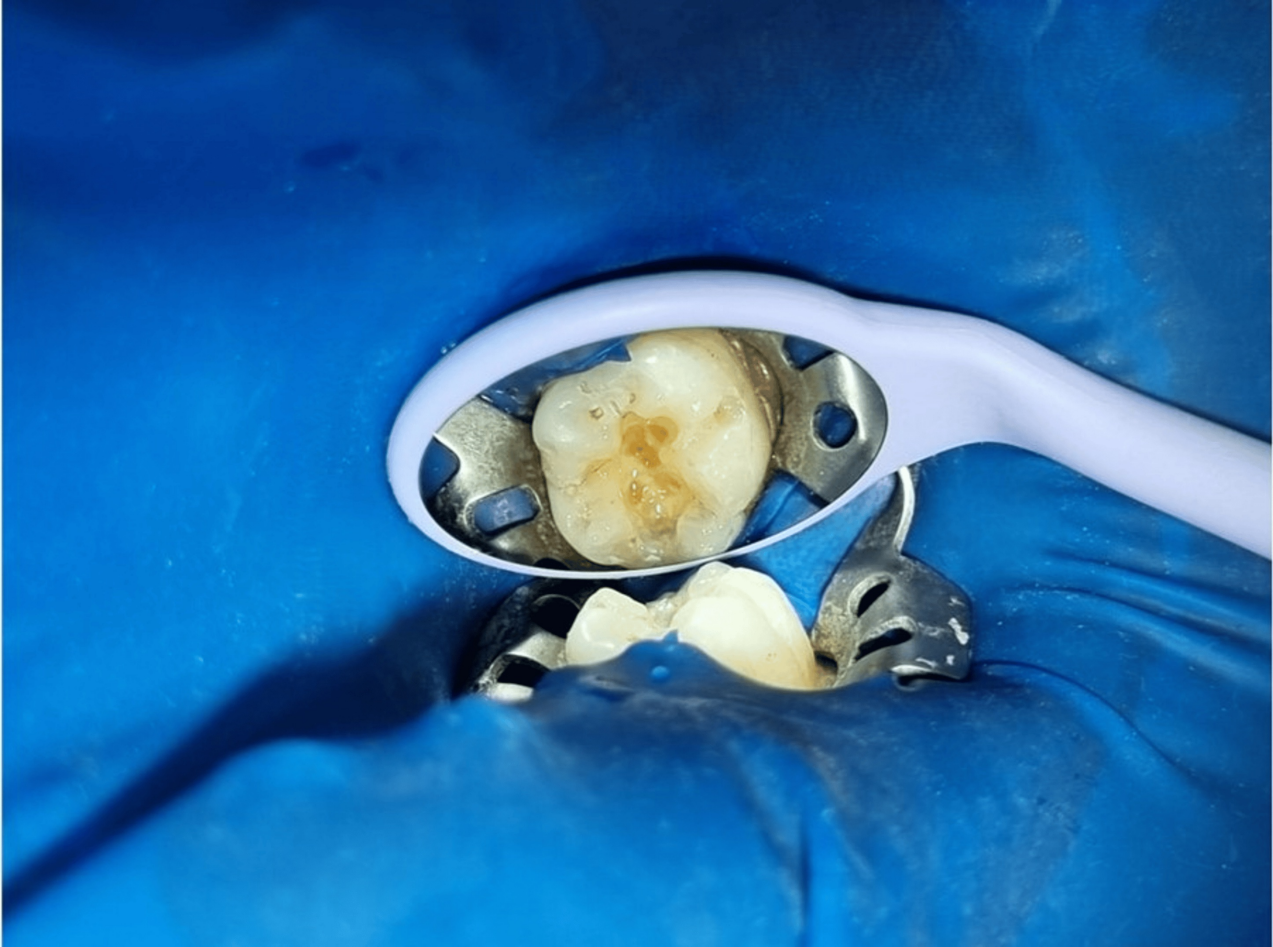
Application of bonding agent

A microbrush was used to apply a single coat of bonding agent (5th Gen, 3M ESPE Single Bond Universal, Minnesota) to the prepared surface, which was polymerized for 20 seconds. The cavity was progressively restored with composite material (Nanohybrid, Spectrum Dentsply Sirona, Charlotte, North Carolina) using the incremental layer technique, keeping 1 mm below the occlusal surface, and then cured for 20 seconds (Figure 6) [1].

**Figure 7:**
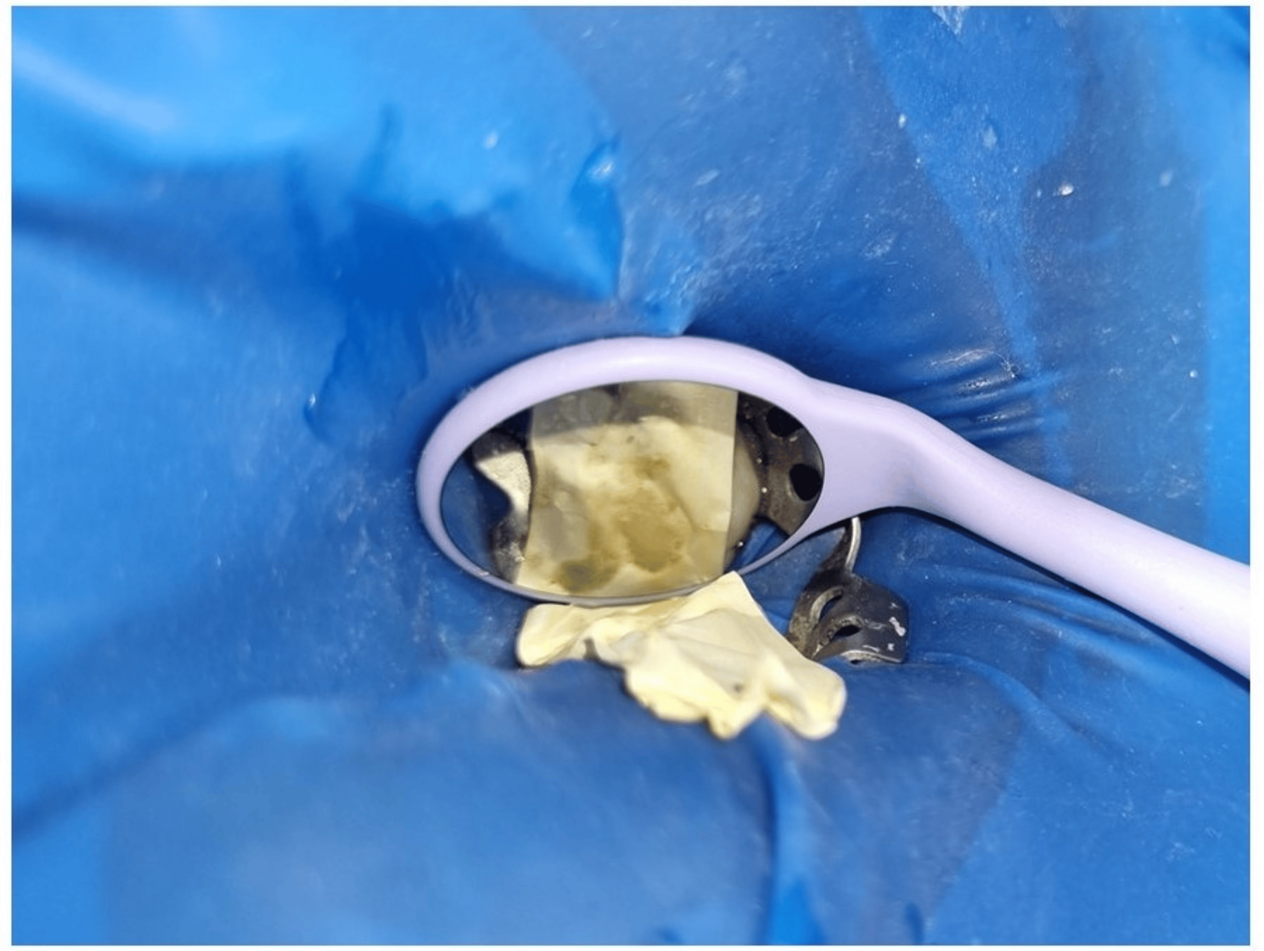
Teflon application over the uncured occlusal surface of the composite

Prior to light curing, the final layer of composite material (Nanohybrid, Spectrum Dentsply Sirona) was applied to the designated area. Subsequently, a section of Teflon tape was carefully put onto the superficial surface of the composite (Figure 7) [2].

**Figure 8:**
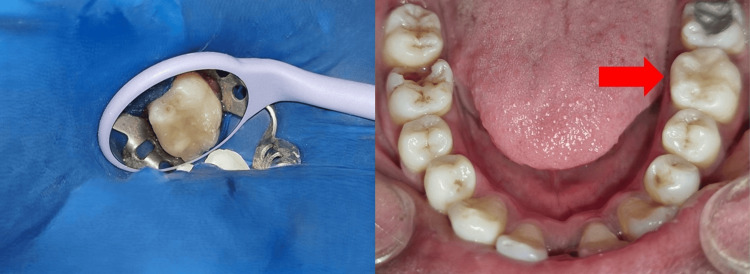
Postoperative photograph of the final composite restoration

Teflon tape is often used in dental procedures for its non-stick properties and ability to create smooth surfaces. After applying Teflon tape, an occlusal stamp and a microbrush were placed on top of the tape. The stamp is a tool used to apply even pressure, making it easier to shape and manipulate the composite material. With the stamp in place, light pressure was gently applied to ensure that the composite material bonded well with the tooth surface and adjacent areas. All excess material on top of the restoration was then removed for a more refined appearance. The next step involved polymerizing the composite material, which hardens and securely adheres to the tooth. This technique typically uses curing light, followed by minimal finishing and polishing with a polishing disc to achieve a smooth surface. This meticulous process ensures that there are no gaps between the composite filling and the tooth structure, resulting in a restoration that has optimal aesthetics and functionality, closely resembling natural teeth and meeting patient needs (Figure 8).

## Discussion

The technique of biomimetic restoration is used to duplicate natural teeth to have a proper occlusion between the adjoining and opposing teeth. This approach aims to provide long-lasting results, improved strength of the remaining tooth structure, and reduced strain on the surrounding structures [2]. There are two main types of biomimetic restoration methods for reducing the pressure exerted during chewing. The first is to eliminate the masticatory strain, which involves removing any factors contributing to excessive force on the teeth, such as adjusting the bite or reshaping the tooth structure. The second is to maximize the tissue-material bond, where the goal is to enhance the bond between the dental material used for restoration (such as composite resin) and the natural tooth structure, ensuring better stability and durability. Increasing bond strength can be achieved through techniques such as proper surface treatment of the tooth, use of bonding agents, and careful placement of composite resin, which leads to a more effective interface between the tooth and restorative material. This strong bond distributes masticatory forces more evenly and prevents micro-leakage, thus enhancing the longevity of the restoration. Studies have shown that these techniques not only improve bond strength but also contribute to the overall durability and performance of the restoration under masticatory forces [4]. In modern dentistry, composite resin is commonly used as a direct restorative material for biomimetic tooth restoration. Researchers from the University of Otago, New Zealand, conducted a study and found that composite resins were used for occluso-proximal (biting surface and side) restorations in premolars 93.7% of the time and in permanent molars 85.2% of the time [5].

The increase in the utilization of composite resins for dental restorations can be attributed to several factors. These include their ability to match the natural color of the teeth, thereby enhancing aesthetics, as well as their capacity for minimal tooth preparation. Additionally, when applied correctly, composite resins exhibit low edge leakage due to effective bonding. They also possess the advantageous qualities of being non-conductive to heat, easily moldable within an extended working period, and capable of one-visit polishing. Furthermore, they offer resistance to fracture and moderate wear, and studies suggest they can endure for approximately eight years before requiring restoration replacement [6,7].

In posterior teeth, carious lesions may develop that do not extend beyond the dentino-enamel junction, indicating caries confined to the enamel and have an intact occlusal morphology. The dentin is destroyed without any damage to the enamel. To access the necrotizing dentine, a considerable amount of healthy enamel must be abraded. This implies that a composite template can be used before the placement of the filling material. Restoration generally aims at getting both form and function [5]. Certainly, if one restores tooth surfaces as they are in reality, then this will increase the patient's loyalty and willingness for dental treatment [1].

The stamp technique is straightforward and feasible for restoring the Class I cavities. When using flowable composite, direct application over the occlusal surface is possible without requiring an isolation agent. However, if deep pits and fissures are present, it is advisable to use an isolation agent. This agent fills the pits and fissures, preventing the flowable composite from seeping inside during application [8]. This approach ensures a smoother, uninterrupted surface for the final restoration. Therefore, it is important to avoid excessive air spraying when applying the isolation agent to the tooth surface. While a flowable composite was used to create the stamp in this instance, various other materials can serve the same purpose, such as gingival barriers [3,9], pit and fissure sealants [3], or transparent acrylic resin [3].

Achieving the accurate occlusal anatomy of a filling is essential for a functional restoration. To counteract the disadvantage of polymerization shrinkage, the restoration is restored by the incremental layer technique. To replicate the natural anatomy of the occlusal surface, a stamp is applied on the final layer, preceded by the placement of Teflon tape as a fence material. Alternatively, cling film can also serve as a barrier material. It is worth noting that the cling film does not need to be removed, as curing can be done through it [1].

Each technique has its pros and cons. The stamp matrix method exerts pressure on the composite, minimizing the formation of microbubbles and interference from oxygen during polymerization [2,10]. An inherent challenge of this technique is that it demands skill and clinical expertise to be executed accurately. It is more commonly employed in cases where preoperative anatomy, particularly in pit and fissure caries, can be preserved, typically in Class I cavities. Since flowable composite is often the material of choice for this method, a decrease in strength can be anticipated. Hence, careful case selection is essential for ensuring successful outcomes when employing this technique. In addition, dentists may focus their office hours on more complex cases, thereby enhancing their reputation [11,1].

## Conclusions

The composite restoration stamp technique is a significant advancement in modern dentistry, providing a precise, efficient, and versatile approach to tooth repair. Using prefabricated templates to shape composite resin materials allows for superior restorations with minimal chairside time, reducing marginal discrepancies and material waste. However, its effectiveness depends on the accuracy of the initial impression and may be limited in complex cases or those requiring extensive tooth structure replacement. Future research should focus on optimizing template materials and improving adaptation to complex occlusal patterns to enhance their clinical utility.
